# Benchmarking seeding strategies for spreading processes in social networks: an interplay between influencers, topologies and sizes

**DOI:** 10.1038/s41598-020-60239-4

**Published:** 2020-02-28

**Authors:** Felipe Montes, Ana María Jaramillo, Jose D. Meisel, Albert Diaz-Guilera, Juan A. Valdivia, Olga L. Sarmiento, Roberto Zarama

**Affiliations:** 1 Department of Industrial Engineering, Universidad de los Andes, Social and Health Complexity Center, Bogotá, Colombia; 2 Facultad de Ingeniería, Universidad de Ibagué, Ibagué, Colombia; 3 Departament de Física de la Matèria Condensada and Universitat de Barcelona Institute of Complex Systems (UBICS), Universitat de Barcelona, Barcelona, Spain; 4 Departamento de Física, Facultad de Ciencias, Universidad de Chile, Santiago de Chile, Chile; 5 School of Medicine, Universidad de los Andes, Social and Health Complexity Center, Bogotá, Colombia

**Keywords:** Epidemiology, Complex networks

## Abstract

The explosion of network science has permitted an understanding of how the structure of social networks affects the dynamics of social contagion. In community-based interventions with spill-over effects, identifying influential spreaders may be harnessed to increase the spreading efficiency of social contagion, in terms of time needed to spread all the largest connected component of the network. Several strategies have been proved to be efficient using only data and simulation-based models in specific network topologies without a consensus of an overall result. Hence, the purpose of this paper is to benchmark the spreading efficiency of seeding strategies related to network structural properties and sizes. We simulate spreading processes on empirical and simulated social networks within a wide range of densities, clustering coefficients, and sizes. We also propose three new decentralized seeding strategies that are structurally different from well-known strategies: community hubs, ambassadors, and random hubs. We observe that the efficiency ranking of strategies varies with the network structure. In general, for sparse networks with community structure, decentralized influencers are suitable for increasing the spreading efficiency. By contrast, when the networks are denser, centralized influencers outperform. These results provide a framework for selecting efficient strategies according to different contexts in which social networks emerge.

## Introduction

Information, behaviors, diseases, emotions, and even the adoption of technological innovations spread through social networks^1–5^. Recently, the explosion of network science across disciplines has produced many important advances in understanding how the structure of social networks affects the dynamics of social contagion. Specifically, the study of social networks has provided an opportunity to potentiate interventions with spill-over effects aimed to increase population well-being. For example, several studies have examined the spreading processes efficiency related to the topological properties of networks^4,6–8^. Other studies have analyzed the role of homophily in spreading processes^9–11^, while others have focused on identifying influential spreaders in networks and how they may be harnessed to increase the efficiency of public health and poverty reduction interventions^12–15^.

A key point for designing interventions with spill-over effects is to allocate resources for the intervention targeting in a wisely way. Thus, it is crucial to have an appropriate methodological framework for selecting seednodes with the best spreading ability. Several complex networks studies have proposed selecting seednodes by ranking network nodes based on centrality measures^12,15–28^. Particularly, nodes with high degree, closeness, and betweenness coefficients have been identified as influential or high-risk individuals during a spreading process^16,23,29^. Furthermore, there are random-walk based seeding strategies, such as Page-Rank, that have been shown more efficient than centrality-based strategies for infecting some networks but less efficient in other ones^19,24,25^. Also, Kitsak *et al*. have proposed that targeting the core of the network by using a K-shell decomposition method is more efficient than targeting central nodes^26^. This approach was later improved by the proposed True core and K-truss decomposition methods^27,28^. Recently, Zhang *et al*. proposed the Vote-Rank decentralized strategy, which seems to experimentally outperform centrality and K-shell methods on both spreading rate and computational efficiency^30^.

Centralized and decentralized seeding strategies have been proved to be efficient using solely data and simulation-based models in specific network topologies without a consensus of an overall result. There is limited evidence on which network structural properties are related to the performance of each seeding strategy. Few studies show that centralized and K-shell based strategies are not efficient in networks with a community structure because chosen spreaders may cluster in the same community or their neighborhoods overlap^18,30^.

We address the gap mentioned above by benchmarking the spreading efficiency of seeding strategies for networks with different structural properties. We simulate spreading processes on a wide range of complex networks, using empirical social networks data, and simulated networks within a range of densities, clustering coefficients, and sizes. We also propose community hubs, ambassadors, and simulated hubs as three new decentralized seeding strategies that are structurally different from those reported by the literature. Our main findings are that the efficiency ranking of the strategies and the degeneracy among strategies differs according to the network structural properties, especially characterized by their density, clustering and size. These results provide a framework for selecting efficient strategies according to different contexts in which social networks emerge.

## Results

**Figure 1:**
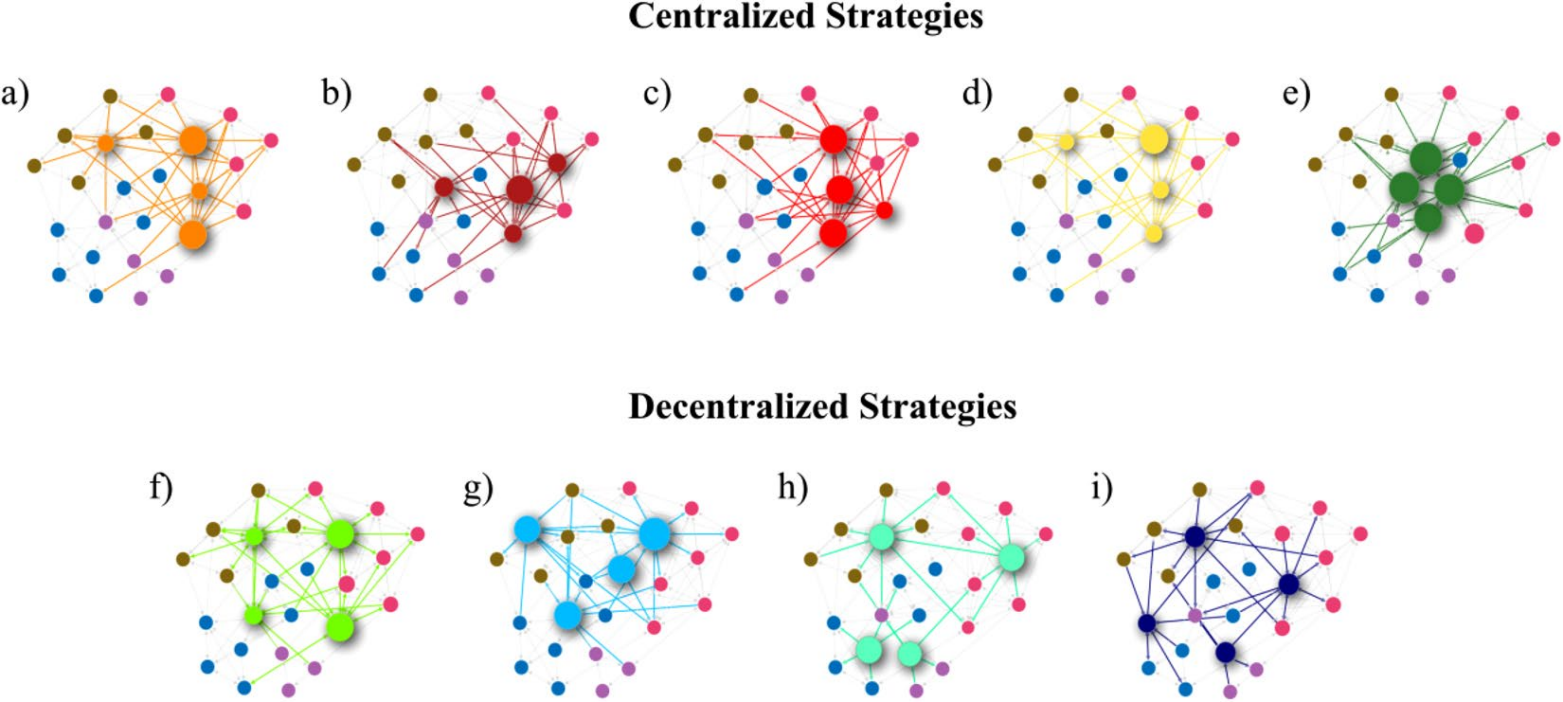
Centralized and decentralized seeding strategies in undirected networks. Nodes color represent communities detected using the Louvain method. The highlighted nodes and their corresponding edges represent the seednodes selected using each strategy, and node size represents the selection order within the seednodes set. Centralized seednodes were those with (**a**) highest degree centrality: Hubs, (**b**) highest betweenness centrality, (**c**) highest closeness centrality, (**d**) highest Page-Rank, and (**e**) nodes in the k-core. Decentralized seednodes are: (**f**) nodes with the highest voting score calculated as the sum of the voting ability of its neighbors: Vote-Rank, (**g**) nodes of a detected community with the highest external degree: Ambassadors, (**h**) nodes of a detected community with the highest internal degree: Community Hubs, and (**i**) the most connected neighbor of randomly chosen nodes: Random Hubs. The Random seeding strategy was not represented in the figure.

We ranked 10 different seeding strategies according to their spreading efficiency. For simplicity, we implemented a susceptible-infected (SI) spreading process^31^ in the largest connected component LCC of five empirical networks and 540 simulated undirected networks with different topologies, seednodes, and sizes. For each scenario, we varied the probability of contagion and the number of seednodes. For ranking the strategies, we calculated the spreading efficiency as the time necessary to infect all nodes of the LCC when starting each contagion from the seednodes. For each network, we initialized the spreading process from 10 different sets of seednodes selected using centralized and decentralized strategies (Fig. 1). Both centralized and decentralized strategies are based on global structural measures and require having data of the full network. Centralized strategies consisted of selecting seednodes with the (a) highest degree centrality: Hubs^3^, (b) highest betweenness centrality^3^, (c) highest closeness centrality^3^, (d) highest Page-Rank^32^; and (e) nodes in the k-core^26,33,34^. Decentralized strategies consisted of selecting (f) nodes with the highest Vote-Rank calculated as the voting score resulting from the sum of the voting ability of the neighbors of each^30^, (g) nodes of detected communities with the highest external degree: Ambassadors, (h) nodes of detected communities with the highest internal degree: Community Hubs, and (i) the most connected neighbor of randomly chosen nodes: Random Hubs. Finally, we measured the spreading efficiency of each strategy for each topology, and we evaluated the degeneracy among strategies (See methods).

For analyzing the results, we categorized each of the empirical and simulated networks according to their topology within three different ranges of density and clustering coefficient. For both measures, our selected ranges were: *Low* from 0 to 0.1, *Medium* from 0.1 to 0.2, and *High* from 0.2 to 1. We categorized the networks within six types (Table 1). Also, we categorized networks according their size as Small with 200 nodes, Medium with 1000 nodes, and Largewith 2000 nodes. We did not simulate larger networks because our focus is to recreate contexts where community-based interventions can be implemented.

**Table 1:** Acronyms of networks structures categorized according to density and clustering coefficient. For example, LD-LC describes a network with low density and low clustering coefficient. We classified the empirical networks in two of these categories, and we generated random networks for the six categories. We represent with * the categories where it is not possible to generate a connected network within the given ranges of density and clustering coefficient. For each category, we generated networks of three sizes: *Small networks* with 200 nodes, *Medium networks* with 1000 nodes, and *Large networks* with 2000 nodes.

		Clustering Coefficient		
		Low [0-0.1]	Medium (0.1.-0.2]	High (0.2-1]
Density	Low [0.-0.1]	LD-LC	LD-MC	LD-HC
	Medium (0.1-0.2]	*	MD-MC	MD-HC
	High (0.2-1]	*	*	HD-HC

### Spreading efficiency for seeding strategies in empirical networks

For measuring the spreading efficiency of the seeding strategies on empirical networks, we ran multiple spreading processes on the largest connected component LCC of five networks representing social systems from different contexts. Ordering from lowest to highest density the networks are: (1) Spanish physicists co-authorships network^35^. (2) Karnataka network: a social network of a rural village in the south of India^12^. (3) Global supply chain project network: an e-mail network between project team roles of a global supply chain project^36^. (4) Recreovia Facebook friendship network: an online friendship network of stakeholders in a physical activity program in Colombia^37^. And (5) School children friendship network: a friendship network of a primary school in Colombia^38^. Networks displayed different topological features, where in their LCC the sizes varied from 25 to 1118 nodes and 87 to 5185 edges. The first two networks were considered of Medium size, and the other three were considered of Small size. The mean degree varied from 3.48 to 22, the densities ranged from 0.004 to 0.15 (the first three networks were in the Low range and the other two were in the Medium range), the clustering coefficient ranged from 0.47 to 0.69 (all of them were in the High range), the average shortest path length ranged from 1.93 to 8.57, and the diameter ranged from 4 to 22 Table 2.

**Table 2:** Characteristics reported for the largest connected component (LCC) of the empirical networks. $N$: Number of nodes, $Ne$: Number of edges, $\delta$: Density, $< C >$: Mean Clustering coefficient, $< k >$: Mean degree, $Nc$: Number of communities, $M$: modularity, $dmax$: diameter of the network, $< d >$: average shortest path length, $r$: degree assortativity coefficient. We ordered networks from lowest to highest density.

Network	$Size(N)$	Ne	$\delta$	$< C >$	$< k >$	$Nc$	$M$	$dmax$	$< d >$	$r$	$Networktype$
Spanish physicists co-authorships network	(Medium) 1162	3017	0.004	0.69	5.19	31	0.9	22	8.57	0.03	*LD-HC*
Karnataka network	(Medium) 1118	5185	0.01	0.68	9.28	25	0.75	9	4.18	0.28	*LD-HC*
Global supply chain project network	(Small) 211	1507	0.03	0.62	7.14	5	0.28	4	2.23	$-$0.28	*LD-HC*
Recreovía facebook friendship network	(Small) 231	2542	0.10	0.52	22	5	0.29	7	2.65	0.08	*MD-MC*
School children friendship network	(Small) 25	87	0.15	0.47	3.48	3	0.33	4	1.93	0.24	*MD-MC*

The simulation results show that, usually, using a seeding strategy is more efficient for initializing a spreading process than randomly selecting the seednodes. However, the efficiency of the strategy depends mainly on the density, clustering coefficient, and size of the network.

**Figure 2:**
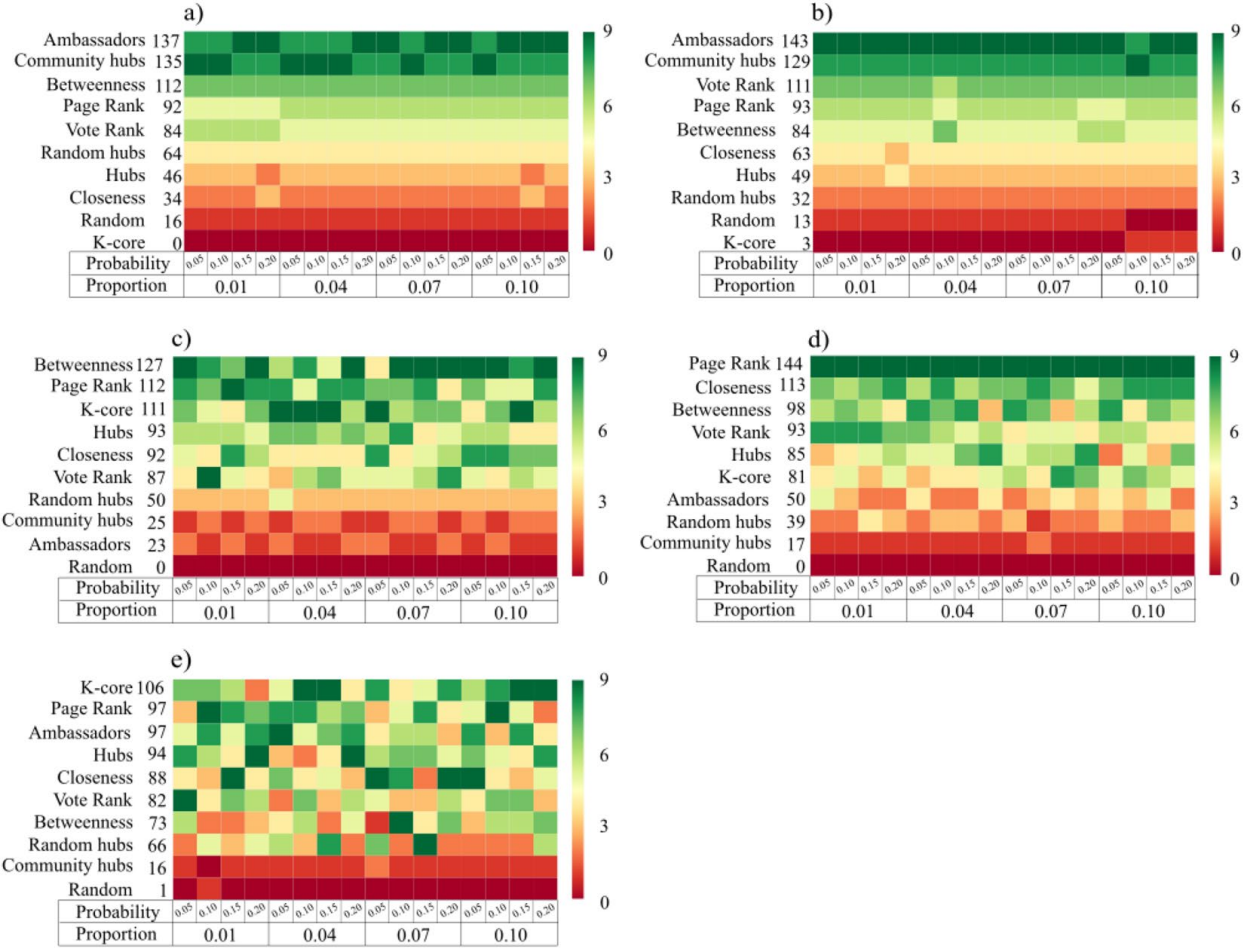
Ranking of the seeding strategies according to their spreading efficiency in five empirical networks by varying the initial percentage of seednodes and the probability of contagion. We ordered the figure panels from the lowest to the highest density of each network: (**a**) Spanish physicists co-authorship network, (**b**) Karnataka network, (**c**) Global supply chain project Network, (**d**) Recreovía Facebook friendship network, and (**e**) School children friendship network. We ranked the 10 seeding strategies according to the number of outperformed strategies in terms of spreading efficiency. We colored as reddest the seeding strategy with less efficiency than the others and as greenest the strategy that outperforms more strategies. The $y$ axis shows the ranking according to the total number of strategies outperformed by each strategy by considering all the different simulation scenarios of the spreading processes.

For Medium networks in the LD-HC category (*Spanish physicists co-authorships network* and *Karnataka network*), the decentralized seeding strategies, Ambassadors and Community Hubs, were the most efficient independently of the probability of contagion $g$ and the number $s$ of seednodes. In terms of spreading efficiency, these strategies were followed in the ranking by the centralized strategies Page-Rank, Betweenness, and the decentralized strategy Vote-Rank. Furthermore, in these networks K-core was the less efficient set of seednodes, even less efficient than choosing seednodes at random. The ranking obtained for these networks is consistent for the different probabilities of contagion and the percentage of seednodes selected (Fig. 2a,b).

Second, we studied our empirical *Small networks*: *Global supply chain project network*, being in the *LD-HC* category, and *Recreovía facebook friendship network, and School children friendship network*, being in the *MD-HC* category. In these networks, the spreading efficiency varied significantly through the different probabilities of contagion and the initial percentage of seednodes. However, we found that the centralized strategy Page-Rank was the most efficient, being in the top two of the ranking for the three networks. Also, contrary to the *Medium networks* of the same structure, the *LD-HC* category, for *Small networks*, Ambassadors and Community Hubs strategies were the least efficient independently of the probability of contagion. Although, these two strategies remained better than randomly selecting the seednodes.

### Spreading efficiency for seeding strategies in simulated networks with different structures

For assessing differences in the spreading efficiency of each seeding strategy according to the network structure, we initialized spreading processes using the 10 seeding strategies in 540 random networks that were distributed in six categories (30 networks per category), and three sizes as explained in Table 1. For preserving skewed degree distributions and small-world properties that were found in the empirical networks, we used an algorithm for growing scale-free networks with tunable clustering^39,41^. We measured structural properties of each type of network (Table 3). We observe that the modularity coefficient does not present a significant variability across realizations within the different network types. Moreover, we observe that on average the modularity coefficient increases when clustering coefficient increases, especially for networks with low density as expected in sparse networks with community structures. We evaluated the seeding strategies in 30 generated networks for each size and type of combinations of density and clustering. For each network we conducted 30 simulations of a particular seeding strategy. The simulation results suggest that the efficiency of each strategy varied depending on the density, clustering coefficient, and size of the networks. Also, we observe that the ranking of strategies changed for each network structure and size, where some results remain consistent depending on the type of seeding strategies, namely, decentralized or centralized.

**Table 3:** Characteristics reported for the largest connected component (LCC) of the generated random networks. Each value is the average of that measure in the 30 generated networks: $Size(N)$: Size of the networks and number of nodes. $Type$: explanation of network Type in the Table 1, $< Ne >$: Average number of edges, $\delta$: Density, $\sigma (\delta )$: Density standard deviation, $< C >$: Average Clustering coefficient, $\sigma (C)$: Clustering standard deviation, $< k >$: Average degree, $Nc[CI-95 \% ]$: Number of communities and confidence interval of $95 \%$ (Values with * don’t have standard deviation), $Q$: modularity, and $\sigma (Q)$: modularity standard deviation.

$Size(N)$	$Type$	$< Ne >$	$\delta$	$\sigma (\delta )$	$< C >$	$\sigma (C)$	$< k >$	$Nc[IC\ 95 \% ]$	$Q$	$\sigma (Q)$
Small (200)	LD-LC	590.97	0.03	$9.0E-06$	0.10	0.017	5.91	9.37 $[\pm 0.34]$	0.37	0.01
	LD-MC	1535.03	0.08	$5.58E-05$	0.16	0.011	15.35	7.60 $[\pm 0.28]$	0.20	0.01
	LD-HC	396.00	0.02	$6.94E-18$	0.40	0.030	3.96	10.57 $[\pm 0.41]$	0.58	0.01
	MD-MC	2253.60	0.11	$7.06E-05$	0.20	0.007	22.54	7.37 $[\pm 0.31]$	0.16	0
	MD-HC	3512.40	0.18	$3.55E-04$	0.31	0.005	35.12	6.40 $[\pm 0.30]$	0.14	0
	HD-HC	9119.60	0.46	$8.52E-04$	0.55	0.004	91.20	3.60 $[\pm 0.25]$	0.06	0
Medium (1000)	LD-LC	19590.93	0.04	$5.80E-06$	0.10	0.002	39.18	9.07 $[\pm 0.36]$	0.13	0
	LD-MC	19411.23	0.04	$2.15E-05$	0.15	0.003	38.82	8.67 $[\pm 0.47]$	0.15	0
	LD-HC	38398.63	0.08	$2.56E-06$	0.43	0.025	76.80	4.07 $[\pm 0.27]$	0.44	0
	MD-MC	56400.00	0.11	$0.00E+00$	0.19	0.002	112.80	8.37 $[\pm 0.26]$	0.07	0
	MD-HC	73585.63	0.15	$1.34E-05$	0.36	0.029	147.17	4.10 $[\pm 0.20]$	0.23	0.03
	HD-HC	181035.50	0.36	$1.56E-04$	0.43	0.001	362.07	2.03 $[\pm 0.07]$	0.11	0.00
Large(2000)	LD-LC	59100.00	0.03	$6.94E-18$	0.08	0.001	59.10	9.43 $[\pm 0.41]$	0.11	0
	LD-MC	96297.00	0.05	$1.33E-05$	0.14	0.001	96.30	7.47 $[\pm 0.28]$	0.11	0
	LD-HC	153588.50	0.08	$2.88E-06$	0.29	0.025	153.59	4.30 $[\pm 0.31]$	0.33	0.04
	MD-MC	225600.00	0.11	$1.39E-17$	0.19	0.001	225.60	8.30 $[\pm 0.32]$	0.05	0
	MD-HC	357568.07	0.18	$2.91E-05$	0.34	0.001	357.57	3.00 *	0.11	0
	HD-HC	494638.50	0.25	$6.39E-05$	0.35	0.001	494.64	3.00 *	0.10	0

**Figure 3:**
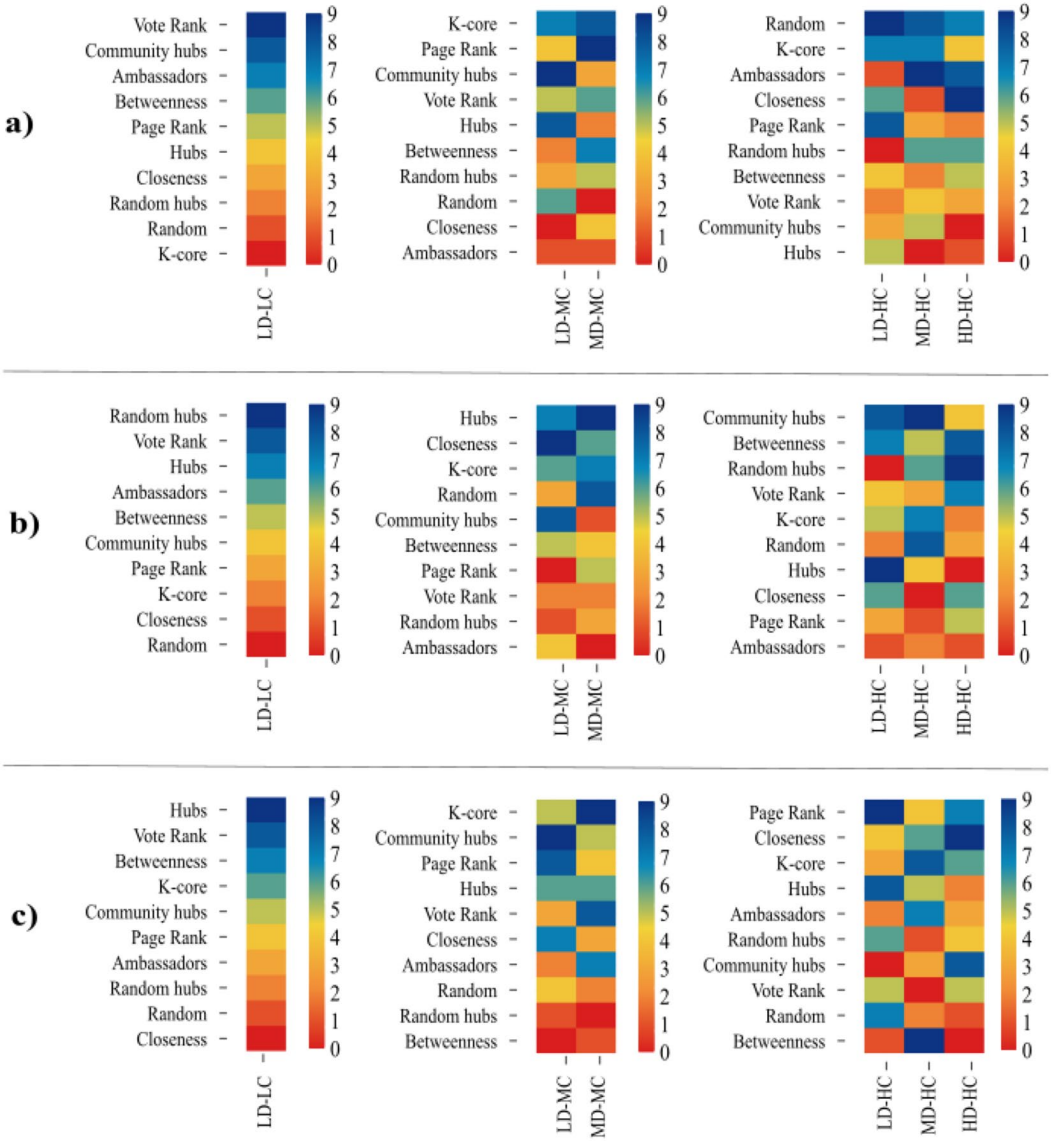
Ranking of the seeding strategies according to their spreading efficiency in 540 random networks classified according to their size, density and clustering. Thirty networks were generated for each combination of size, density, and clustering ranges using an algorithm of growing networks with tunable clustering^39^. Each panel represents a network size: (**a**) *Small networks* (200 nodes), (**b**) *Medium Networks* (1000 nodes), and (**c**) *Large Networks* (2000 nodes). Within each panel, networks structures are shown with acronyms according to the ranges of density and clustering coefficient as explained in Table 1, and ordered from left to right according to their clustering coefficient range. For each network category, we ranked the 10 seeding strategies according to the number of outperformed strategies in terms of spreading efficiency. The ranking was obtained by adding the number of strategies outperformed by each strategy in 30 spreading processes simulation runs for each one of the 30 networks of that category. Then, the heatmap was obtained according to the ranking by coloring reddest the seeding strategy with zero outperforms, and bluest the strategy with nine outperforms. For each network category, strategies were ordered according to the ranking from top to bottom. For initializing the spreading process, we fixed the number of seednodes as the number of detected communities in each network using a the Louvain method for community detection^40^.

In the case of decentralized seeding strategies, the spreading efficiency was higher when networks were in the *LD-LC* category, independently of the network size. For this particular network structure, within the decentralized strategies, the ranking varied according to network size: (a) *Small networks*: Vote-Rank, Community-Hubs, and Ambassadors (Fig. 3 panel (a); (b)) *Medium networks*: Random-Hubs, Vote-Rank, and Ambassadors (Fig. 3 panel (b); (c))*Large networks*: Vote-Rank (Fig. 3 panel (c)). Nevertheless, independently of the size in *LD* networks, as clustering coefficient increases to 0.2 (*MC*), the only decentralized strategy that remains efficient is Community-Hubs. For *MD-HC* in *Small* and *Large networks*, Ambassadors remains efficient, while Community-Hubs is the most efficient strategy in *Medium networks*. In addition, for *HD-HC*, Ambassadors strategy is efficient in *Small networks*, Random-Hubs in *Medium networks*, and Community-Hubs in *Large networks*.

In the case of centralized seeding strategies, the spreading efficiency was higher when networks had medium or high density and clustering coefficient (0.1–1]. In those cases, independently on network size, K-Core was consistently efficient among other centralized strategies in *MD-MC* and *MD-HC* networks. Furthermore, Page Rank strategy was efficient for *Small networks* in *MD-MC* and *LD-HC*. In the case of *Large networks*, Page Rank was efficient in *LD-HC* and *HD-HC* categories. In addition, Closeness strategy was consistently efficient for *Small* and *Large networks* in *HD-HC*.

In general, the performance of decentralized vs. centralized strategies, as groups of strategies, does not depend on network size. Moreover, we observe that three particular strategies are consistently in the top three most efficient regardless of the network size: (1) The decentralized strategies Vote-Rank and Community-Hubs are top ranked for networks with low density and low or medium clustering (*LD-LC* and *LD-MC*), respectively, and (2) the centralized strategy K-Core is top ranked for networks with medium density and medium or high clustering (*MD-MC* and *MD-HD*) (Table 4). Besides, we found that for networks with extreme connectivity or extremely segregated clusters (*HD-HC* and *LD-HC* networks, respectively) rankings are not consistent for different sizes.

**Table 4:** Summary of the top three most efficient strategies ranked for each combination of density, clustering, and size of the networks. Network structures are shown with acronyms according to the ranges of density, clustering coefficient and size as explained in Table 1. Network size is represented by S: *Small networks (200 nodes)*, M: *Medium networks (1000 nodes)*, and L: *Large networks (2000 nodes)*. Strategies are ranked from 1 (most efficient strategy) to 3.

Type	Size	Centralized					Decentralized				Random
		Hubs	Betweenness	Closeness	Page-Rank	K-Core	Vote-Rank	Ambassaddors	Community-Hubs	Random-Hubs	
LD-LC	S						1		2	3	
	M	3					2			1	
	L	1	3				2				
LD-MC	S	2				3			1		
	M	3		1					2		
	L			3	2				1		
LD-HC	S				2				3		1
	M	1	3						2		
	L	2			1						3
MD-MC	S		3		1	2					
	M	1				3					2
	L					1	2	3			
MD-HC	S					3		1			2
	M					3			1		2
	L		1			2		3			
HD-HC	S			1				2			3
	M		2				3			1	
	L			1	3			2			

Nevertheless, when analyzing more in-depth the efficiency of each particular strategy, we observe that the ranking varies according to network size. We calculated the standard deviation for the density and clustering coefficient for the 30 realizations of every network type and size (Table 3). We observe that the four types of networks (*LD-LC*, *LD-MC*, *MD-MC*, and *MD-HC*) that have more consistent results in the ranking are those with the lower clustering coefficient variability. We also observe that the standard deviation of density is always lower than $1.56\times 1{0}^{-4}$, so we discarded that the variation in density causes differences in the ranking. However, we observe that the results were not consistent for the different sizes of the two types of networks that exhibit the highest variability in clustering coefficient and density (*LD-HC* and *HD-HC*, respectively). Our hypothesis is that decentralized strategies could be efficient for *LD-HC* networks due to their community structure explained by a high value of modularity, and that centralized strategies were suitable for the *HD-HC* networks due to their high connectivity. Nevertheless, those hypotheses were rejected for these types of networks with extreme values of density and clustering in their structures.

Our previous results of the most efficient strategies for each network type remain consistent when considering the modularity and number of communities as metrics for determining the community structure of the network types. The decentralized strategies, Vote-Rank and Community-hubs are efficient regardless of the network size for *LD-LC* and *LD-MC* types, which have higher modularity values and number of communities. Also, the centralized K-core strategy is in the top three regardless of size for networks with lower modularity values and fewer communities, such as the *MD-MC* and *MD-HD* networks. Also, we did not find consistent results for different sizes of networks with extreme values of modularity and number of communities: (1) *LD-HC* networks have the highest modularity, and (2) *HD-HC* networks have one of the lowest number of communities and modularity values. We could hypothesize that in *LD-HC* networks, the decentralized strategies are not consistently efficient as the small number of edges between different communities could be encapsulating the spreading processes inside the seednodes’ communities avoiding an inter-community spreading. On the other hand, in the *HD-HC* networks, the centralized strategies are not consistently efficient due to the high connectivity of the network that could lead to a low differentiation among seednodes sets.

As in empirical networks, in most of the topologies and sizes of simulated networks, using a strategy for selecting seednodes was more efficient than choosing the seednodes at random. However, in *Small networks* when the clustering coefficient was high (0.2–1] choosing the seednodes at random remained efficient (Fig. 3 panel (a), third sub-panel).

### Degeneracy among seednodes

The same node may belong to different sets of seednodes. Thus, to better understand the results observed in the spreading efficiency rankings, we evaluated the degeneracy among each pair of seeding strategies. We define the degeneracy coefficient of two sets of seednodes (not to confound with k-degeneracy used in graph theory) as the fraction of nodes that belong to both sets. Let A and B two sets, $Degeneracy\,(A,B)=| A\cap B|$/$| A\cup B|$. For each network size and topology, we calculated the average degeneracy coefficient among each pair of seeding strategies over the 30 simulated networks.

**Figure 4:**
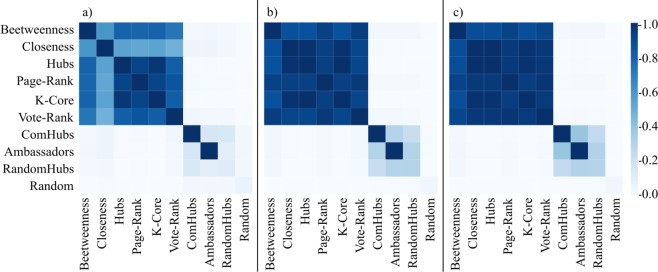
Degeneracy coefficient of seeding strategies for 30 simulated networks in the *LD-LC* category. Each panel represents a network size: (**a**) *Small networks* (200 nodes), (**b**) *Medium networks* (1000 nodes), and (**c**) *Large Networks* (2000 nodes). We define the degeneracy coefficient of two sets of seednodes (not to confound with k-degeneracy used in graph theory) as the fraction of nodes that belong to a pair of sets. Let A and B two sets, $Degeneracy\,(A,B)=| A\cap B|$/$| A\cup B|$. The lighter blue indicates a 0 of degeneracy coefficient between a pair of strategies, meaning that the two strategies did not have common nodes. The darker blue, as in the diagonal, indicates a degeneracy coefficient of 1, meaning that both strategies contain the same nodes.

We observe that the degeneracy coefficient shows a pattern that remains similar for the different networks and topologies (Fig. 4). We observe that all centralized and Vote-Rank strategies shared, on average, more than 50% of nodes independently of the network size. In the case of the decentralized strategies, the proportion of common nodes with other strategies ranged from 20% to 40% for different network sizes, showing a higher diversification of the seednode selection compared to the centralized strategies. Furthermore, independently of density, clustering coefficient, and network size; the degeneracy among centralized and decentralized strategies was low. The result for the *LD-LC* category in the three network sizes is shown in Fig. 4 as an example of the general pattern observed in the different network sizes and topologies.

## Discussion

This study provides a benchmark for selecting efficient strategies for initializing interventions with spill-over effects in social networks with different structures. Our main finding is that the efficiency of each seeding strategy depends on the network structure, particularly on the density and clustering coefficient. In general, for sparse networks with community structure, Community-hubs, which are decentralized influencers, are suitable for increasing the spreading efficiency. By contrast, when the networks are denser, nodes in the K-core, which are centralized influencers, outperform. We observe also that, usually, independently on the network structure, having a strategy for selecting seednodes for a spreading process is better than using random sampling. This result is critical for providing evidence to improve commonly used random sampling methods for delivering interventions. Also, our results are coherent with studies that have shown the importance of homophily and community structure of networks for understanding the spread and adoption of behaviors^9,42^.

As a first result for both empirical and simulated networks, we found that the decentralized strategy Community Hubs remained efficient for *Medium networks* in the *LD-HC* category. *LD-HC* networks are likely to have a community structure, therefore decentralized strategies allow to identify seednodes in the different communities and avoiding a potential overlap among the seednodes dyads. This leads to an increase in the coverage range of the spreading process by taking advantage of the weak ties as spreading channels between communities^43,44^. The importance of avoiding overlap in networks with community structure while selecting a seeding strategy might explain that, for the *LD-HC* category, in *Medium* and *Large* networks sizes, and in both empirical and simulated networks, K-core is not an efficient strategy. The reason is that K-core seednodes are likely to have a high number of overlapping neighbors causing a reduced coverage of susceptible nodes, at least at the initial steps of the spreading process. Similar reasons might be suggesting that central seeding strategies, such as Closeness strategy, does not perform as well as decentralized strategies when density is low. Central nodes have been also shown to be connected by strong ties to other network actors, increasing their overlapping relationships^44^. Employing decentralized strategies might be desirable in real contexts with sparse or segregated populations. In those settings, conducting searches for identifying local leaders, Community Hubs, might be more convenient for performing direct and indirect influence than conducting searches for identifying influential individuals at the population level^45^. Hence, using the Community Hubs strategy can potentiate the effect of community-based interventions, by reinforcing individual perceptions and behavioral changes, as Community-Hubs strategy facilitates to conduct customized processes within each community detected^46^. Also, Community Hubs could be used as an alternative to the recently proposed Vote-rank strategy, especially when access to the entire network data is limited or unavailable, Vote-Rank cannot be calculated.

As a second general result for both empirical and simulated networks, we found that the centralized seeding strategy Page Rank remained efficient for *Small networks* in the *MD-MC* and *LD-HC* categories. For different network sizes, K-Core seeding strategy performs efficiently when density is in the medium range, and clustering coefficient increases, i.e. the *MD-MC* and *MD-HC* categories. In fact, due to the network medium density, nodes with high Page-Rank and nodes in the K-Core are likely to be directly connected to nodes in different areas of the network. Denser networks are not likely to display community structures, and having a medium or high clustering coefficient implies that overlap among nodes is high. Thus, decentralized strategies are not likely to add more coverage than centralized strategies. This can be evidenced by the low spreading efficiency obtained by Ambassadors and Community hubs in the categories *MD-MC* and *MD-HC* for *Small* and *Medium* network sizes. Employing centralized strategies might be desirable in contexts with dense and cohesive populations. In those settings, identifying global leaders for delivering interventions might be more efficient than conducting local searches in communities that are not well defined.

For simulated networks, we found that Vote-Rank seeding strategy remained efficient for the *LD-LC* category of networks in the three network sizes. *LD-LC* networks are likely to have a larger shortest path length than the other topologies studied due to their low connectivity. Thus, this result is coherent with previous studies where the Vote-Rank strategy was more efficient when the shortest path length among seednodes was larger^30^.

Of course, this study has some limitations. First, we used a simulation-based approach to calculate the spreading efficiency of different strategies. The above might bias the results to specific network topologies and spreading conditions. However, we aimed to build different scenarios by considering a wide range of probabilities of contagion, number of seednodes, and networks with different topologies and sizes. Second, for simplicity, we used the susceptible-infected model for the simulations assuming a cascade process for the contagion and different results may emerge using other spreading processes. Nevertheless, for this work it was important to compare the different strategies with the same and most straightforward model to avoid confounding on the efficiency between the spreading process dynamics and the seednodes selection. Although, we consider that future work should explore different spreading methods. Third, we generated networks to simulate social networks with skewed degree distributions and small-world properties^41^; hence, our results might not apply to other situations where networks have other degree distributions.

Identifying influential individuals for the design of interventions has been of interest to practitioners and researchers due to its effect on delivering successful and cost-efficient interventions at the community level. Our results provide a first outlook to selecting efficient strategies for allocating resources during behavioral interventions with spill-over effects in different contexts, and in terms of centralized and decentralized strategies. Future work should address more detailed explanations on common features and possible causes of the different rankings at the seednodes sets level within and between centralized and decentralized strategies.

## Methods

We propose a simulation-based approach for ranking ten centralized and decentralized seeding strategies for initializing a spreading process according to their spreading efficiency. First, we conduct the ranking for five empirical networks with different topologies and sizes. Then, we simulated specific network structures to observe possible associations among structural properties and the seednodes spreading efficiency. We categorized each one of the empirical and simulated networks within three different ranges of both density and clustering coefficient as explained in Table 2.

### Network characteristics

We gather data from five empirical networks to evaluate the spreading efficiency of the seeding strategies. We calculated structural measures of the largest connected of these empirical networks, and we listed the information regarding those measures in Table 1). We consider social networks of different contexts. (1) Spanish physicists co-authorships network: a collaboration network built from the American Physical Society, which covers scientific collaborations between Spanish physicists between 2010 and 2012^35^. In this network, nodes represent researchers and edges represent co-authorship. We categorized it as *LD-HC Medium network*. (2) Karnataka network: a social network built from village 19 in Karnataka, India for the diffusion of a microfinance program conducted by the Abdul Latif Jameel Poverty Action Lab in 2006^12^. In this network, nodes represent individuals, and an undirected tie connects two nodes if one of the individuals reported at least one of 12 types of relationships related to trust. We categorized it as *LD-HC Medium network*. (3) Global supply chain project network: an email network between project team roles of a global supply chain project^36^. This network is an approach to project management where team members belong to different organizations of the supply chain, located in more than one geographic location and time zone, and contribute to different phases of a project. In this network, the nodes represent team members, and directed edges represent the different emails sent and received by the project team members to coordinate and implement the different activities. We categorized it as *LD-HC Small network*. (4) Recreovía facebook friendship network: an online friendship network of stakeholders in a physical activity program in Colombia. This program aims to promote physical activity, health habits, and social equity through musicalized and directed group classes in Bogota, Colombia^37^. In this network, nodes represent Facebook friends of the Recreovia account, and edges represent a mutual friendship between the nodes. Our research group built the Recreovia friendship network in 2016 for analyzing social cohesion emerging from the program. We categorized it as *MD-MC Small network*. (5) School children friendship network: a friendship network of one school classroom where nodes represent children, and directed edges represent friendship nominations^38^. We collected data from the Colombian site of the International Study of Childhood Obesity, Lifestyle, and Environment (ISCOLE); a collaborative study conducted in schools of 12 countries.^47^ We categorized the network as *MD-MC Small network*.

### Spreading efficiency for seeding strategies in empirical networks

#### The susceptible-infected spreading model

For each network, we simulate the spreading process using the cascade susceptible-infected: SI model, where the spreading driver is interaction^3^. In this model, each susceptible node may become infected depending on their infected neighbors^31,48^ and infected nodes cannot recover. At the time $t=0$, all network nodes are susceptible except for a set of seednodes that are infected. We consider the probability of infection $g$ constant and equal for every infected node. At every time step, for each susceptible node, we randomly choose one of its neighbors for interacting. If the selected neighbor is infected, then the susceptible node will become infected with a probability $g$ and will remain susceptible with a probability $1-g$. If the neighbor is susceptible, nothing happens. We set the number of seednodes fixed for four proportion values: 0.01, 0.04, 0.07, and 0.10. The process is repeated for each time step until all the network LCC is infected. We determined the spreading efficiency of each seeding strategy as the time needed to infect all the LCC of the network, starting the spreading from those seednodes.

#### Seeding strategies

We compared ten seeding strategies: five centralized, four decentralized, and one random for identifying seednodes based on structural properties of each network (Fig. 2).

Centralized strategies consist of selecting nodes with (a) Highest degree centrality defined as the highest number of edges adjacent to a node^3^. (b) Highest Betweenness centrality defined as the highest frequency of appearance of a node in the shortest paths between all the pairs of nodes of the network^3^. (c) Highest Closeness centrality defined as the lowest average shortest path length from a node to all the other nodes of the network^3^. (d) Highest Page-Rank defined as the highest probability that a random walker visits the node^32^. And (e) nodes selected from the k-core of the network using a k-shell decomposition algorithm^26,33,34^.

For decentralized strategies, first, we applied the Louvain algorithm to detect communities maximizing modularity^40,49^. Then, we selected: (f) Nodes of detected communities with the highest external degree: Ambassador. (g) Nodes of detected communities with the highest internal degree: Community Hub. (h) Nodes with the highest voting score calculated as the sum of the voting ability of its neighbors: Vote-Rank. The voting ability for each node in the network represents the number of votes that the node can provide to its neighbors^30^. (i) the neighbor with the highest degree of randomly chosen nodes (Random Hubs). Finally, we also selected random seednodes (Random).

To build seednodes sets with equal size, for each centralized and decentralized strategy, we assigned a set of a fixed number $s$ of seednodes equal to the number of communities detected in each network. For each of the centralized-based seednodes, we selected the $s$ nodes with the highest respective centrality measure. In case that several nodes had the same centrality measure, we randomly selected the necessary number of $s$ seednodes. For the k-core seednodes, we randomly selected $s$ nodes in the k-core of the network. If $s$ was higher than the k-core size, we randomly selected the remaining nodes in the (k-1)-core. For the decentralized strategies Ambassadors and Community Hubs, we sort in descending order the communities according to their size. Then, we selected one Community Hub or Ambassador per community. We repeated the process until $s$ nodes were selected. For random seednodes, we chose $s$ nodes at random.

### Spreading efficiency for simulated networks

For analyzing the relationship among the strategies of seednodes and the structure of the network, we generated 30 simulated networks for the six topologies and the three different sizes (Table 1). We used an algorithm of growing scale-free networks with tunable clustering^39^, so that it preserves skewed degree distributions and small-world properties of social networks used in this manuscript^41^. The algorithm builds networks of a fixed number of nodes, and connects them following a preferential attachment behavior until a desirable density is reached, as in the traditional Barabasi-Albert model^50^. Then, it incorporates triad formation among one of the connected nodes of every new edge until achieving a desirable clustering coefficient. We show structural properties of each type of network in 3, where each value is the average of that measure in the 30 generated networks. After generating each network, we ran 30 times the SI spreading process, initializing from each strategy, and infecting all the network nodes. We ranked the seeding strategies by taking into account the spreading efficiency, i.e. the time needed to infect the entire LCC of the network, obtained while infecting 30 networks, with 30 runs for each network. For each run, we calculated the number of seeding strategies that each strategy outperformed, in terms of spreading efficiency. Then for each combination of clustering coefficient and density, we summed the efficiency score for each strategy over the 30 runs and the 30 networks. Finally, we ranked the strategies based on the total scores obtained. Strategies in the top of the ranking have a value of 9, meaning that they outperform the other nine strategies over the 900 instances. By contrast, the strategy at the bottom of the ranking has a value of 0, meaning that it does not outperform any other seeding strategy.

#### Degeneracy Coefficient among seednodes

In order to better understand the results observed in the spreading efficiency rankings, we evaluated the degeneracy among each pair of sets of seednodes. We define the degeneracy coefficient of two sets of seednodes (not to confound with k-degeneracy used in graph theory) as the proportion of seednodes shared by both strategies over the total number of nodes of both strategies. Let A and B two sets of seednodes, $Degeneracy(A,B)=| A\cap B|$/$| A\cup B|$. When degeneracy coefficient equals 1 between a pair of sets of seednodes, it means that both sets contain the same nodes, while degeneracy coefficient equals 0, it means that that both sets of seednodes are entirely composed by different nodes.
